# Cardiovascular function and psychological distress in urbanised black South Africans: the SA BPA study

**DOI:** 10.5830/CVJA-2010-022

**Published:** 2010-08

**Authors:** N Mashele, JM Van Rooyen, L Malan, JC Potgieter

**Affiliations:** School for Physiology, Nutrition and Consumer Sciences, North-West University, Potchefstroom Campus, Potchefstroom, South Africa; School for Physiology, Nutrition and Consumer Sciences, North-West University, Potchefstroom Campus, Potchefstroom, South Africa; School for Physiology, Nutrition and Consumer Sciences, North-West University, Potchefstroom Campus, Potchefstroom, South Africa; School for Psychosocial Behavioural Sciences, North-West University, Potchefstroom Campus, Potchefstroom, South Africa

**Keywords:** depression, perception of health, cardiovascular function, urbanised Africans, hypertension

## Abstract

**Objective:**

The increased prevalence of cardiovascular disease risk factors in sub-Saharan Africa has increased the incidence of cardiovascular disease in this region but whether psychological distress contributes to this observed increased risk remains largely unclear.

The aim of this study was to investigate the association between cardiovascular function and psychological distress in urbanised black South African men (*n* = 101) and women (*n* = 99).

**Methods:**

Resting cardiovascular variables were obtained by making use of the Finometer device and 24-hour ambulatory blood pressure (BP) measurements with the Cardiotens apparatus. Psychological questionnaires assessed the perception of health (General Health questionnaire) and depression status (DSM-IV criteria). The resting ECG (NORAV PC-1200) was used to determine left ventricular hypertrophy (LVH) by making use of the Cornell product. Confounders included age, obesity, alcohol intake, smoking and physical activity.

**Results:**

The hypertensive groups were overweight, with lower vascular compliance and higher LVH (only men) compared to the normotensive groups. In hypertensive men, perception of health (somatic symptoms) was positively associated with blood pressure, while in hypertensive women it was associated with heart rate. Major depression was associated with LVH in hypertensive men and mean arterial pressure in hypertensive women. LVH and depression showed odds ratios of 1.02 (95% CI: 0.997–1.05) and 1.15 (95% CI: 1.01–1.32), respectively, in predicting hypertension in women.

**Conclusions:**

Psychological distress was associated with higher blood pressure in hypertensive African men but also with the development of left ventricular hypertrophy in hypertensive African men and women.

## Summary

Cardiovascular disease (CVD) is one of the leading causes of death worldwide, with the greatest mortality occurring in low-and middle-income countries.^1^ In black Africans, CVD has been associated with an inherent salt sensitivity, low-renin hypertension and urbanisation.^2^-^4^

It remains largely unclear, though, whether the experience of psychological distress may contribute to the observed increase in CVD risk. With increasing environmental demands, such as experienced in urbanised areas, the inability to adapt or cope may manifest as behavioural (e.g. substance abuse, dietary changes, inactivity), psychological (e.g. depression and psychosomatic complaints) and medical (cardiovascular functioning and other physical illnesses) consequences.^5^,^6^-^11^ The effect of known confounders for CVD risk^12^ should therefore at least be acknowledged in this novel psycho-physiological approach.

Additionally, the loss of social and cultural support, which often accompanies urbanisation, may lead to psychosocial disruption and an associated increase in psychological distress. This may contribute to the high incidence of hypertension in urban black Africans.^11^ In this study, self-report questionnaires on participants’ perception of their own health and depression^13^,^14^ were used to gain insight into the level of psychological distress experienced by them. A thorough description of these measures will be provided in the methods section.

Other studies have shown a relationship between depression and CVD, such as coronary heart disease (CHD) and coronary artery disease.^15^,^16^ Unfortunately, these studies focused on the role of depression in CVD post-cardiac event. Conflicting results were found with regard to depression and the development of hypertension in the African-American population. Shinn *et al*.^17^ found that their results did not support the character of depressive symptoms in the development of hypertension in normotensive adults.^17^ Other researchers found that the association between depression and the risk of hypertension compared favourably with better-established predictors of hypertension, such as obesity.^18^

To our knowledge, investigations exploring the association between psychological well being or functioning and CVD have not been done in the African context. Therefore, the aim of this study was to investigate whether there is a relationship between cardiovascular function and psychological distress in hypertensive and normotensive urbanised black Africans of the North West province of South Africa.

## Methods

The methods for this study were adapted and abbreviated from the Sympathetic Activity and Ambulatory Blood Pressure in Africans (SABPA) study.^19^ The SABPA study was a multidisciplinary target-population study conducted in 2008. Included in the data collection were urbanised black Africans from governmental organisations in the North West province. During recruitment, two months prior to data collection, the protocol was explained to each participant and they were given an opportunity to ask questions. Thereafter informed consent was obtained.

Urbanised black African individuals (101 men and 99 women) between 25 and 60 years of age who complied with the inclusion criteria of having the same socio-economic status (SES) and work environment were included. The exclusion criteria were: pregnancy, lactation, high temperature (> 37°C), users of α- and β-blocking agents, users of psychotropic agents, blood donors or having been vaccinated in the past three months before taking part in the study.

The participants were stratified into two groups: hypertensive (HT) and normotensive (NT) men and women. This stratification was done according to the European Society of Hypertension (ESH) 2007 guidelines where average 24-hour ambulatory hypertensive status is defined as systolic and/or diastolic blood pressure (≥ 125–130/≥ 80 mmHg).^20^

The study was approved by the Ethics Committee of the North-West University, Potchefstroom Campus, in accordance with ethical guidelines of the World Medical Association’s Declaration of Helsinki.^21^

## Experimental procedure

The experimental procedure for each participant followed a two-day protocol. On the first day, the Cardiotens device (Meditech CE0120®) was applied and programmed to record the 24-hour BP of four participants, one each day of the working week. The physical activity meter was fitted around the waist and a physical activity (GPAIQ) questionnaire was completed at school. Thereafter, the four participants left to resume their normal daily activities. The Cardiotens device recorded BP measurements in 30-minute intervals during the daytime and in 60-minute intervals during the night.

At approximately 16:40 the participants were transported to the Metabolic Unit research facility of the North-West University (research unit for human studies) where they stayed overnight. The unit is well equipped with 10 furnished bedrooms, a kitchen, two bathrooms and a dining area. The procedures for the evening included a brief introduction to the apparatus to minimise the ‘white-coat effect’,^22^ and a tour of the facilities. Completion of the psychosocial battery of questionnaires followed, under supervision of registered psychologists and fieldworkers. The questionnaires were arranged in such a way as to reduce the effects of participant fatigue, with half the questionnaires being completed before dinner, and the remaining half thereafter. The participants had dinner at 18:00 and enjoyed their last beverages between 20:00 and 22:00 (tea/coffee and biscuits) before going to bed at 22:00.

The procedure for day two included the disconnection of the Cardiotens apparatus at 06:00. After obtaining the anthropometric measurements, the participants were taken to the blood pressure station where the cardiovascular measurements were obtained while the participants were in a semi-Fowlers position. The same procedures were followed for the rest of the participants daily.

## Demographic questionnaire

Included in the demographic questionnaire were questions on smoking and alcohol consumption. These are self-report questions with a ‘yes’ or ‘no’ answer. Information on physical activity levels was obtained with the Global Physical Activity questionnaire.^23^ This measures the total physical activity participation in three domains: (1) activity at work, (2) travel to and from places and (3) recreational activities. The sum of these domains were then evaluated and summed in calories per week. Physical activity is classified as high (vigorous-intensity activity on at least three days, achieving a minimum of 1 500 METS-minutes/week, or seven or more days of any activity accruing at least 3 000 METS-minutes/week) or low (not meeting any of the above criteria).^23^

## Psychological questionnaires

The 28-item GHQ^13^ is used for the assessment of signs and symptoms of psychological dysfunction and is useful for understanding various sources of distress in occupational research.^24^ The GHQ is a measure of common mental health and focuses on symptom domains for depression, anxiety, somatic complaints and social withdrawal.^23^

The GHQ was validated for use within the Setswana-speaking population prior to the study.^25^ An example of items used in this questionnaire include: ‘Have you found everything getting on top of you?’, ‘Have you been getting scared or panicking for no reason?’ and ‘Have you been getting edgy and bad tempered?’. Each of the above are then accompanied by four possible responses; ‘not at all’, ‘no more than usual’, ‘rather more than usual’ and ‘much more than usual’. In this study each item was evaluated using the binary scoring method.^26^

The two least symptomatic answers are given a score of nil (0) while the two most symptomatic answers are given a value of one (1). Total scores exceeding the threshold of four are classified as achieving ‘psychiatric caseness’. In general practice, individuals classified as achieving ‘psychiatric caseness’ would be likely to receive further attention.^25^ The reliability coefficients of the subscales for this questionnaire varied between 0.77 and 0.83.

The Patient Health questionnaire (PHQ) is a sensitive nineitem instrument for making criteria-based diagnosis of depressive disorders and it is also a reliable and valid measure of severity of depression.^14^ The scale is based on the actual nine criteria of diagnosis of the DSM-IV depressive disorders. The PHQ assesses diagnoses divided into threshold disorders (disorders that correspond to specific DSM-IV diagnoses, i.e. major depressive disorder) and sub-threshold disorders (disorders whose criteria include fewer symptoms than required for any specific DSM-IV diagnoses, i.e. other depressive disorders). The questionnaire scores each of the nine DSM-IV diagnostic criteria as being experienced ‘0’ (not at all) to ‘3’ (nearly every day).

For analysis, the PHQ-9 scores are divided into the following categories of increasing severity: 0–4, 5–9, 10–14, 15–19 and 20 or greater, which represent minimal, mild, moderate, moderately severe, and severe depression, respectively. Scores less than five signify the absence of depressive disorders, scores of 5–9 predominantly represent no depression or sub-threshold depression, scores of 10–14 represent a spectrum of individuals who may or may not display depression, and scores of 15 or higher usually are indicative of major depression.^14^ In this study, scores of ≤ 10 were considered as the absence of depression (MDD = 0) and values > 10 were considered as the presence of major depression (MDD = 1).^14^ The Cronbach alpha-reliability index for this sample was 0.81.

## Anthropometric measurements

All measurements were standardised and taken in triplicate to the nearest 0.1 cm by registered biokineticists. Height was measured by making use of a stadiometer while the participant’s head was in the Frankfurt plane.^27^ Weight was measured to the nearest 0.1 kg using a Krups scale with the participants wearing minimal clothing. These measurements were used for the calculation of body mass index (body mass/height^2^).^28^ Physical activity was measured using an Actical® accelerometer (Montréal, Québec).^29^ Waist circumferences were measured with a metal tape at the midpoint between the lower costal border and the iliac crest, perpendicular to the long axis of the trunk.^27^

## Cardiovascular measurements

A Cardiotens apparatus (Meditech CE0120®) was used for the 24-hour ambulatory blood pressure measurement (SBP and DBP) and a 12-lead ECG (Norav PC-1200) was applied to obtain six resting cardiac cycles. Non-invasive rested continuous arterial blood pressure recordings were obtained for five minutes using the Finometer device (Finapres Medical Systems, Amsterdam, the Netherlands). The Fast Modelflo computer software programme analysed the results to provide: mean arterial pressure (MAP) and total peripheral resistance (TPR), arterial compliance (Cw) and heart rate (HR). Left ventricular hypertrophy (LVH) was calculated from the 12-lead ECG device using the following gender-specific formula:

Cornell product: sum of all the leads, (RaVL + SV3 ≥ 2.8 mV in men and ≥ 2.0 mV in women)* QRS > 244 ms.^30^

## Statistical analysis

All data were analysed by means of the computer software package STATISTICA 8 (StatSoft, Inc., Tulsa, OK, USA, 2008). All data were normally distributed, hence parametric methods were used. A single 2 × 2 × 2 (hypertension × depression × gender) analysis of covariance (ANCOVA) was done to evaluate the main effects interactions for cardiovascular and psychological distress data. Subsequent 2 × 2 ANCOVA (24-h HT × depression) in men and women and (gender × depression) analyses followed. The prevalence of smoking, alcohol consumption, hypertension, hypertension medication, physical activity (PAI) and depression were computed using the two-way Pearson Chi-square analysis. One-way ANCOVA was used to compare the psychological and cardiovascular variables between the hypertensive and normotensive gender groups while independent of confounders (age, BMI, PAI, smoking and alcohol consumption).

Partial correlations followed to indicate associations between cardiovascular variables (WC, Cw, MAP, 24-h SBP and the Cornell product), depression (PHQ-9) and the common mental health domains of depression (GHQ_DS), anxiety (GHQ_AS), somatic symptoms (GHQ_SS) and social dysfunction (GHQ_SD), as well as the GHQ_Total score (GHQ_T) separately in the hypertensive men and women. Partial correlations were done while adjusting for confounders (age, BMI, PAI and smoking and alcohol consumption).

Logistic regression analysis was done using hypertension as the dependent variable and GHQ_SS, PHQ_TT, Cornell product, blood pressure, MAP and vascular compliance as the predictor variables. The odds ratio was determined to measure effect size. The reliability of GHQ-28 and PHQ-9 were determined by the Cronbach alpha (α) reliability coefficient, which was between 0.77 and 0.83 for GHQ-28 and 0.81 for PHQ-9. Data were considered statistically significant at *p* ≤ 0.05.

## Results

The 2 × 2 × 2 (24-h HT × depression × gender) interactions were not significant for either of the cardiovascular variables, depression and perception of health data. For exploratory reasons, cardiovascular variables were evaluated in subsequent two-way ANCOVAs, (24-h HT × depression) in men and women, which showed significance for own perception of own health [GHQ-T (F: 1, 91) = 3.98, *p* = 0.05; GHQ-AS (F: 1, 91) = 4.02, *p* = 0.05] and GHQ-DS, [(F: 1, 91) = 4.17, *p* = 0.05]. The 2 × 2 (gender × depression) interaction showed a significant interaction for LVH [(F: 1, 163) = 7.30, *p* = 0.01].

Table 1 shows that more men were hypertensive (79%) than women (57%). The hypertensive men and women were older (*p* = 0.01), more obese (*p* < 0.01), and with larger waist circumference (WC) (*p* = 0.05) compared with their normotensive counterparts. The hypertensive groups also revealed a higher Cornell product value only in HT men (*p* = 0.06) coupled to a lower arterial compliance (*p* = 0.05), compared with the normotensive groups.

**Table 1.** Descriptive Statistics, Mean (95% CI) Of The Hypertensive And Normotensive Men And Women Independent Of Confounders (Age, BMI, Smoking, Alcohol And Physical Activity)

	*Hypertensive men (n = 79)*	*Normotensive men (n = 21)*	p*-value*	*Hypertensive women (n = 57)*	*Normotensive women (n = 42)*	p*-value*
*Age (years)	**44.39 (42.61; 42.17)**	**39.10 (35.64; 42.55)**	**0.01**	46.61 (44.58; 48.65)	43.74 (41.37; 46.11)	0.07
*BMI (kg/m^2^)	**28.48 (27.24; 29.73)**	**23.10 (20.65; 25.48)**	**< 0.01**	**34.63 (32.71; 36.54)**	**30.38 (28.16; 32.61)**	**< 0.01**
Waist circumference (cm)	**100.6 (92.15; 109.07)**	**82.13 (65.74; 98.53)**	**0.05**	**99.59 (95.90; 103.27)**	**85.52 (81.23; 89.81)**	**< 0.01**
*Smoking *n* (%)	26 (34.21)	5 (23.80)	0.42	3 (5.26)	0 (0)	0.13
*Alcohol n (%)	34 (43.04)	6 (28.57)	0.23	7 (12.28)	5 (11.90)	0.95
*PAI *n* (%)	56 (70.88)	15 (71.43)	0.80	41 (71.93)	34 (80.95)	0.27
HIV+ *n* (%)	12 (15.19)	2 (9.53)	0.20	4 (7.02)	1 (2.38)	0.30
Hypertension medication *n* (%)	14 (17.72)	1 (4.76)	0.14	15 (26.31)	6 (14.29)	0.15
TPR (mmHg/ml/s)	1.07 (0.04; 1.00)	1.05 (0.895; 1.21)	0.80	0.99 (0.90; 1.09)	0.92 (0.80; 1.03)	0.29
MAP (mmHg)	**113.34 (110.12; 115.85)**	**101.53 (95.48; 107.58)**	< 0.01	**105.68 (103.41; 107.95)**	**95.60 (92.88; 98.31)**	**< 0.01**
C_W_ (ml/mmHg)	**1.84 (1.77; 1.92)**	**2.03 (1.87; 2.19)**	0.05	**1.74 (1.66; 1.81)**	**2.01 (1.92; 2.10)**	**< 0.01**
Heart rate (b/min)	68.20 (65.73; 70.68)	63.90 (58.67; 69.12)	0.16	70.39 (67.10; 73.69)	68.82 (64.87; 72.78)	0.56
Cornell product (mV.ms)	**89.65 (78.29; 101.02)**	**60.02 (32.44; 87.61)**	0.06	60.53 (53.36; 67.71)	49.58 (40.98; 5820)	0.65
24-h DBP (mmHg)	**91.48 (88.68; 92.63)**	**76.92 (72.80; 81.04)**	< 0.01	**84.25 (82.47; 86.03)**	**72.38 (70.28; 74.48)**	**< 0.01**
24-h SBP (mmHg)	**142 (138.84; 144.56)**	**122.61 (116.65; 128.58)**	< 0.01	**137.10 (134.21; 139.91)**	**117.37 (114.01; 120.73)**	**< 0.01**
PHQ_TT	8.33 (7.07; 9.6)	8.31 (5.72; 10.90)	0.99	10.48 (8.92; 12.02)	10.12 (8.30; 11.95)	0.80
GHQ_T	7.21 (5.74; 8.68)	8.20 (5.13; 11.25)	0.58	9.23 (7.41; 11.04)	9.10 (6.93; 11.21)	0.91
GHQ_Somatic symptomsS	2.38 (1.91; 2.84)	2.35 (1.38; 3.32)	0.97	2.60 (1.95; 3.25)	2.74 (1.97; 3.51)	0.79
GHQ_Anxiety symptomsS	2.31 (1.77; 2.86)	2.60 (1.46; 3.72)	0.67	2.77 (2.10; 3.50)	3.10 (2.28; 3.90)	0.57
GHQ_Social dysfunctionD	1.70 (1.20; 2.20)	2.21 (1.18; 3.23)	0.39	2.44 (1.85; 3.01)	1.94 (1.25; 2.62)	0.29
GHQ_Depressive symptomsS	0.82 (0.48; 1.17)	1.03 (0.31; 1.76)	0.62	1.43 (0.84; 2.01)	1.30 (0.60; 1.97)	0.75

CI, 95% confidence intervals; *n*, number of participants; BMI, body mass index; PAI, physical activity index; TPR, total peripheral resistance; MAP, mean arterial pressure; C_W_, arterial compliance; 24-h SBP, 24-hour systolic blood pressure; 24-h DBP, 24-hour diastolic blood pressure; PHQ_TT, patient health questionnaire total score; GHQ_T, general health questionnaire total score; Statistical significance is considered when, *p* ≤ 0.05. Significant values are highlighted in bold. *not adjusted.

Table 2 reveals that in HT men, blood pressure (systolic, *r* = 0.24 and diastolic, *r* = 0.30) was associated with perceived health (GHQ_SS) and target end-organ damage (LVH). LVH (*r* = 0.32) was positively associated with depression whereas it was negatively associated (*r* = –0.26) with vascular compliance in HT men. In the HT women, HR correlated positively with the perception of somatic symptoms (*r* = 0.30). To determine the effect size of these associations in predicting hypertension, a logistic regression analysis was performed. Hypertension was used as the dependent variable and SBP, DBP, Cw, LVH, GHQ_SS and PHQ_TT were independent predictors. In only the women, depression and LVH showed an odds ratio of 1.15 (95% CI = 1.01–1.32) and 1.02 (95% CI = 0.998–1.05), respectively, as predictors of hypertension.

**Table 2.** Partial Correlations In Hypertensive African Men And Women: Cardiovascular Variables With Depression (PHQ-9), Perception Of Somatic Symptoms And Target End-Organ Damage (LVH) Independent Of Confounders (Age, BMI, Smoking, Alcohol And Physical Activity)

	*Hypertensive men*			*Hypertensive women*		
	*Target end-organ damage (LVH) r-value; p-value*	*Perception of somatic symptoms r-value; p-value*	*PHQ_major depression r-value; p-value*	*Target end-organ damage (LVH) r-value; p-value*	*Perception of health (GHQ_SS) r-value; p-value*	*PHQ_major depression r-value; p-value*
Waist circumference (cm)	–0.02; 0.86	0.13; 0.30	–0.18; 0.13	0.22; 0.06	0.04; 0.73	–0.16; 0.18
MAP (mmHg)	**0.44; < 0.01**	0.18; 0.12	0.15; 0.21	0.20; 0.08	–0.14; 0.22	**0.23; 0.05**
Hear rate (b/min)	0.03; 0.81	0.15; 0.22	–0.05; 0.69	–0.13; 0.27	**0.30; 0.01**	–0.03; 0.83
TPR (mmHg/ml/s)	0.13; 0.26	0.10; 0.41	0.10; 0.38	0.18; 0.11	–0.20; 0.08	0.05; 0.69
C_W_ (ml/mmHg)	**–0.26; 0.03**	-0.15; 0.20	–0.12; 0.32	–0.10; 0.37	0.11; 0.35	–0.18; 0.13
SBP (mmHg)	**0.43; < 0.01**	**0.24; 0.04**	–0.00; 1.00	0.21; 0.06	–0.07; 0.55	0.05; 0.69
DBP (mmHg)	**0.32; 0.01**	**0.30; 0.01**	–0.01; 0.97	0.12; 0.31	–0.11; 0.34	0.11; 0.35
Cornell product (mV.ms)	–	–0.07; 0.56	**0.35; < 0.01**	–	0.03; 0.83	–0.10; 0.40

LVH, left ventricular hypertrophy; BMI, body mass index; PAI, physical activity index; MAP, mean arterial pressure; HR, heart rate; TPR, total peripheral resistance; C_W_, arterial compliance; SBP, systolic blood pressure; DBP, diastolic blood pressure; Cornell product (> 2.44 mV.ms). PHQ_ major depression, scores of < 10 are considered as showing no depressive disorders; whilst a score >10 are considered to be major depressed. All cardiovascular variables were adjusted for age, BMI, PAI, smoking and alcohol consumption. Significant correlations are highlighted in bold.

## Discussion

The main aim of this study was to investigate the interaction between cardiovascular function and psychological distress in urbanised black South Africans. The main findings of this study were that in HT men, blood pressure (SBP and DBP) was associated with the perception of more somatic symptoms and LVH, while LVH was also associated with depression (*p* = 0.001). In HT women, HR was associated with the perception of more somatic symptoms and MAP was associated with depression. Additionally, this study showed that in HT African men, elevated BP and lower vascular compliance were both associated with the development of target end-organ damage.

Based on the DSM-IV depression criteria as measured with the PHQ-9, more women revealed a trend of experiencing depression compared to men, which is consistent with results found in African-Americans.^31^ When comparing the five levels of severity of depression, however, a new trend emerged. While women were more prone to suffer from minimal (6%) to moderately severe (36%) depression, substantially more men (28%) seemed to suffer from severe depression in comparison to women (15%). These differences may be due to the discrepancies in the expression of depression by men and women. The expression of emotions, constrained by traditional notions of masculinity, may explain why the prevalence of depression was high in men even though they did not report symptoms of depression.^32^

African men had a higher prevalence of hypertension compared to the women, which was consistent with other studies done on this population group.^4^,^8^-^11^ If participants in this study were stratified into hypertensive and normotensive groups, significant associations existed between the perception of somatic symptoms and 24-hour BP in the HT men. This finding suggests that individuals who suffer from high BP have a negative experience of their own physical health, and that they are aware of being physically not well. It has previously been found that increased BP manifests among Africans with a negative perception of their well being.^11^

This is also consistent with findings from other studies that showed that Africans may experience more chronic sympathetic system activation when exposed to social and environmental stressors.^8^,^10^ The same authors illustrated that in African men, an exaggerated peripheral resistance response could be seen.^4^,^10^,^11^ The perception of daily events as stressful might result in a negative experience of physical health, psychological distress and perceived poor health. These experiences may manifest as subjective stress, resulting in an exaggerated vascular response and subsequent increases in BP.^33^

Regarding the cardiovascular profile, our data revealed a practical significance for lower arterial compliance predicting HT (odds ratio 9.75), but it was also positively associated with the development of target end-organ damage.^34^,^35^ The cardiovascular profile in urban African men was additionally associated with depression. As 28% of them were severely depressed, a vicious circle seems apparent and CVD risk could be increased if psychological distress or depression persists.^36^ It has been shown that hypothalamic–pituitary–adrenocortical (HPA) axis hyperactivity has been associated with both hypertension and depression. Whether HPA hyperactivity is a possible mechanism for the above associations in this population remains an unsubstantiated speculation that will require further investigation.^37^-^39^

A logistic regression analysis was also performed to show the effect size of depression, perception of health and cardiovascular variables as predictors for HT. Other studies have shown that in men, depression has been significantly associated with a variety of cardiovascular disorders, particularly the elevation of MAP.^17^,^40^,^41^ In this study, though, a weak association between depression and MAP was found only in hypertensive women. Depressed women were 1.15 times more likely to develop hypertension than men, indicating that depression had a greater effect on HT in women than the other measured predictors. Therefore, individuals who were depressed had a greater chance of developing hypertension. As was found in African-Americans, depression was predictive of later incidence of hypertension.^42^

The possible reasons for this discrepancy in findings may lie in the difference in the populations under study. Different backgrounds, socio-economic status, living conditions and levels of stress and depression may be additional confounders for comparing studies in different settings. Moreover, the use of different psychological models in diagnosing depression may result in an incongruity in the sensitivity of the instruments.

Lastly, the urban African men revealed a 24-hour mean BP of 138/89 mmHg, which was higher than the ESH recommendations (> 125–130/> 80 mmHg), suggesting a possible need for new cut-off values (24-hour AMBP) for Africans. On the other hand, it could also indicate the seriousness of uncontrolled BP in urban black African men experiencing increased psychological distress, because only 16% of the HT men used antihypertensive medication and 79% conformed to the ESH criteria of hypertensive status. We therefore recommend that pre-hypertensive levels should be monitored and addressed with early non-pharmacological lifestyle modifications.

Possible limitations of the study include the small size of the study sample when subjects were divided in HT and NT groups. Future research should incorporate the measure of psychological well being in addition to the measure of psychopathology, as it will provide a broader continuum for the classification of the mental health of those individuals falling within the threshold category. The lack of symptoms of depression in a certain part of the current sample should therefore not be interpreted as the presence of mental health.

## Conclusion

This study showed that depression was significantly associated with certain measured cardiovascular variables and that depression was the most prominent contributor to HT. Major depression was associated with the development of pathological conditions such as the development of LVH, lower vascular compliance and elevated MAP, possibly through hyperactivity of the sympathetic nervous system. Perception of poorer health, in particular somatisation, could contribute to autonomic dysfunction in both men and women. The limited number of similar studies in an African population serves as motivation for more research in this area.
